# Before *PAX1*, *DKK1*, and D-loop variants are held responsible for the Chiari-I malformation, the pathogenicity of each variant must be proven

**DOI:** 10.3325/cmj.2026.67.50

**Published:** 2026-02

**Authors:** Josef Finsterer

**Affiliations:** Department of Neurology, Neurology and Neurophysiology Center, Vienna, Austria

To the Editor,

I read with interest the case study by Rosdi et al reporting on a 15-year-old girl with Chiari I malformation (C1M), which was attributed to previously undescribed variants in the D-loop of mtDNA (m.210A>G, m.16031C>G, m.16145G>A, m.16181A>G) in the *PAX1* (c.556 G>A) and *DKK1* genes (c.548-3T>C) (1). The authors proposed a causal link between these variants and C1M (1). The study is noteworthy, but several points should be discussed.

The first point is that the four homoplasmic variants in the D-loop are most likely not pathogenic and therefore have no impact on the clinical picture of the index patient. Mitochondrial diseases due to mtDNA variants are usually multisystemic diseases affecting the central nervous system, eyes, ears, endocrine organs, heart, viscera, and kidneys (2). None of these mtDNA variants has yet been described as pathogenic. A further argument against the pathogenicity of any of these variants is that they all occur in the homoplasmic state. Pathogenic mtDNA variants normally occur in the heteroplasmic state. Another argument is that, in 75% of cases, mtDNA variants are inherited through the maternal line (3). Classification of a mtDNA variant as pathogenic requires not only documentation of heteroplasmy but also segregation of the biochemical defect in individual muscle fibers with a high heteroplasmy rate, segregation with the phenotype within a family, biochemical studies documenting the biochemical defect, and cybrid studies (4). Since these criteria were not met in the index study, the reported mtDNA variants should be classified as benign. Another argument against pathogenicity is that four different variants together are usually not causative for a specific phenotype.

The second point refers to the *PAX1* variant c.556 G>A and the *DKK1* variant c.548-3T>C (1). The *PAX1* and *DKK1* variants were not associated with CIM. The discovered *PAX1* and *DKK1* variants did not alter the protein structure of the gene product. Functional analyses, segregation analyses of large pedigrees, and inclusion of large control groups, necessary to assess the potential pathogenicity of *PAX1* and *DKK* mutations, were not performed (5).

In summary, this interesting study has limitations that affect the results and their interpretation. Addressing these limitations could strengthen the conclusions and support the message of the study. Before *PAX1*, *DKK1*, and D-loop variants are held responsible for C1M, the pathogenicity of each variant in these genes needs to be established.
